# Off-label use of dexmedetomidine in paediatric anaesthesiology: an international survey of 791 (paediatric) anaesthesiologists

**DOI:** 10.1007/s00228-020-03028-2

**Published:** 2020-10-29

**Authors:** Camille E. van Hoorn, Robert B. Flint, Justin Skowno, Paul Davies, Thomas Engelhardt, Kirk Lalwani, Olutoyin Olutoye, Erwin Ista, Jurgen C. de Graaff

**Affiliations:** 1 Department of Anaesthesiology, Erasmus University Medical Centre -Sophia Children’s Hospital, Rotterdam, The Netherlands; 2 Department of Paediatric Surgery, Erasmus University Medical Centre -Sophia Children’s Hospital, PO Box: 2060, 3000 CB Rotterdam, The Netherlands; 3 Division of Neonatology, Department of Paediatrics, Erasmus University Medical Centre -Sophia Children’s Hospital, Rotterdam, The Netherlands; 4 Department of Hospital Pharmacy, Erasmus University Medical Centre, Rotterdam, The Netherlands; 5 Department of Anaesthesiology, Children’s Hospital at Westmead, University of Sydney, Sydney, Australia; 6 Department of Anaesthesia and Pain Management, The Royal Children’s Hospital, Melbourne, Australia; 7 Department of Anaesthesia, Royal Children’s Hospital Aberdeen and School of Medicine, University of Aberdeen, Aberdeen, UK; 8 Department of Anaesthesia, McGill University Health Center, Montreal Children’s Hospital, Montreal, QC Canada; 9 Department of Anaesthesiology and Paediatrics, Oregon Health and Science University, Portland, OR USA; 10 Department of Anaesthesiology, Peri-operative and Pain Medicine, Texas Children’s Hospital, Houston, TX 77030 USA; 11 Department of Internal Medicine - Nursing Science, Erasmus University Medical Centre, Rotterdam, The Netherlands; 12 Department of Paediatric Surgery, Paediatric Intensive Care, Erasmus University Medical Centre - Sophia Children’s Hospital, Rotterdam, The Netherlands

**Keywords:** Anaesthesia, Paediatrics, Dexmedetomidine, Pharmacology, Drug prescriptions, Off-label use

## Abstract

**Purpose:**

The purpose of this international study was to investigate prescribing practices of dexmedetomidine by paediatric anaesthesiologists.

**Methods:**

We performed an online survey on the prescription rate of dexmedetomidine, route of administration and dosage, adverse drug reactions, education on the drug and overall experience. Members of specialist paediatric anaesthesia societies of Europe (ESPA), New Zealand and Australia (SPANZA), Great Britain and Ireland (APAGBI) and the USA (SPA) were consulted. Responses were collected in July and August 2019.

**Results:**

Data from 791 responders (17% of 5171 invitees) were included in the analyses. Dexmedetomidine was prescribed by 70% of the respondents (ESPA 53%; SPANZA 69%; APAGBI 34% and SPA 96%), mostly for procedural sedation (68%), premedication (46%) and/or ICU sedation (46%). Seventy-three percent had access to local or national protocols, although lack of education was the main reason cited by 26% of the respondents not to prescribe dexmedetomidine. The main difference in dexmedetomidine use concerned the age of patients (SPA primarily < 1 year, others primarily > 1 year). The dosage varied widely ranging from 0.2–5 μg kg^−1^ for nasal premedication, 0.2–8 μg kg^−1^ for nasal procedural sedation and 0–4 μg kg^−1^ intravenously as adjuvant for anaesthesia. Only ESPA members (61%) had noted an adverse drug reaction, namely bradycardia.

**Conclusion:**

The majority of anaesthesiologists use dexmedetomidine in paediatrics for premedication, procedural sedation, ICU sedation and anaesthesia, despite the off-label use and sparse evidence. The large intercontinental differences in prescribing dexmedetomidine call for consensus and worldwide education on the optimal use in paediatric practice.

**Supplementry Information:**

The online version of this article (10.1007/s00228-020-03028-2) contains supplementary material, which is available to authorized users.

## Introduction

Dexmedetomidine is increasingly used in children for premedication, sedation in the intensive care unit (ICU), procedural sedation and anaesthesia, but also to prevent postoperative agitation, nausea and vomiting [1, 2]. Dexmedetomidine is an alpha-2 adrenoceptor agonist that provides adequate sedation with facilitated arousal and analgesia without respiratory depression. Dexmedetomidine offers advantages over traditional anaesthetics for its hemodynamic stability, sedative properties and multimodal pain management [3, 4]. Its colourless and odourless properties make it suitable for paediatric intranasal administration as premedication. Furthermore, dexmedetomidine ameliorates separation anxiety in children [5, 6].

Despite the lack of evidence and off-label use of dexmedetomidine for anaesthesia in patients younger than 18 years of age, the worldwide use of dexmedetomidine for paediatric anaesthesia is still increasing [7, 8]. Experimental research has shown that dexmedetomidine may have a neuroprotective effect when co-administered with other anaesthetic medications [9, 10]. However, the evidence is lacking from clinical studies and randomized controlled trials on the short- and long-term effects of dexmedetomidine use in children undergoing general anaesthesia or receiving prolonged dexmedetomidine infusions. Multiple ongoing studies in children investigate the relationship between dexmedetomidine-based general anaesthesia and long-term neurodevelopmental outcomes [4, 11].

Furthermore, dosing protocols for children have not yet been published, and an international consensus on the use of dexmedetomidine in paediatrics is missing [12–16]. Incorrect application could lead to yet unknown adverse long-term effects. Therefore, overexposure to the drug should be avoided.

We postulated that dexmedetomidine is frequently used in paediatric anaesthesia without a structured implementation procedure including, for example, education and protocols, which leads to large application differences. We performed a survey of international paediatric anaesthesia specialist societies in order to gather information on the use of dexmedetomidine in children by paediatric anaesthesiologists worldwide and identify areas of future need for safe and effective use of dexmedetomidine in children.

## Methods

We performed an online survey on the use of dexmedetomidine in paediatric anaesthesiology, starting July 16, 2019, and with the closing date August 16, 2019. The target response rate was 25%.

Survey respondents were recruited from the following societies for paediatric anaesthesiologists: European Society for Paediatric Anaesthesiology (ESPA), Society for Paediatric Anaesthesia in New Zealand and Australia (SPANZA), Association of Paediatric Anaesthetists of Great Britain and Ireland (APAGBI) and Society for Paediatric Anesthesia (SPA). The boards of these societies were invited to distribute a survey, described below, amongst their members via an e-mail with a link to the survey. ESPA, APAGBI and SPA sent an e-mail with the link to the survey to all members. SPANZA mentioned the survey and link in a newsletter. ESPA and SPANZA sent a reminder 1 month later. APAGBI and SPA have e-mail usage protocols for survey distribution in place, which do not provide for reminders.

### Survey

Experts in the field of paediatric anaesthesia developed a 16-question survey to collect information on the use of dexmedetomidine in paediatric anaesthesiology care. The items addressed whether the respondent regularly prescribes dexmedetomidine to children, perceived barriers for the use of dexmedetomidine in practice, in what setting dexmedetomidine was used, the availability of a local protocol, how the respondent had been educated on dexmedetomidine in paediatrics and clinical experiences with the drug, such as adverse events (survey in Supplementary Data).

The survey was designed to be completed anonymously within 10 min by every anonymous participant. It was delivered through Limesurvey (Limesurvey GmbH, Hamburg, Germany) secure web application for building and managing online surveys and databases [17]. The survey was tested amongst paediatric anaesthesiologists at the Erasmus MC-Sophia Children’s Hospital Rotterdam. Following this test, some of the questions were adjusted for improvement.

### Analysis

The survey data were exported from Limesurvey to Microsoft Excel Version 16.32 (Microsoft Corporation, Redmond, Washington, USA) and SPSS Statistics Version 24 (IBM Corporation, Armonk, NY, USA) to perform descriptive statistical analysis. SPSS was used to compare the groups and summarize the data. Due to the explorative nature of the study, no comparative statistics were performed.

Incomplete responses with at least 40% completion were still included for analysis. The numbers of respondents per question were determined in order to provide accurate response rates per question.

Responses of respondents who had not stated the country of practice were excluded from analysis because these responses could not be assigned to a societal group.

Questions asked regarding experience with adverse drug reactions were not specified as to whether these reactions were experienced once or that these were experienced more frequently.

## Results

**Fig. 1:**
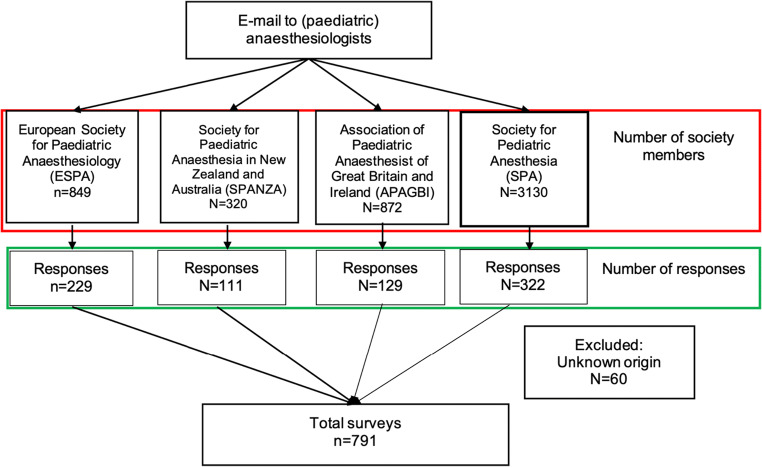
Distribution and response diagram of the survey

In total, 5171 society members received an invitation (Fig. 1). The number of anaesthesiologists who were members of multiple societies was unknown. Sixty respondents were excluded because the country of practice was missing. The overall response was 17% and varied from 35% (SPANZA) to 10% (SPA) amongst the various societies (Table 1). Seventy-five incomplete surveys were included for analysis. No incomplete surveys were excluded due to > 40% completion of the survey. Respondents who did not answer a specific question were left out of consideration regarding this question, as reflected in Tables 1, 3 and 4. Across all societies, most respondents worked in a tertiary hospital as a full-time paediatric anaesthesiologist (693/791, 88%). Respondents had practised as a physician for a median duration of 12 years [IQR 6–21].

Almost all (96%) SPA respondents used dexmedetomidine in paediatric practice (310/322) versus 69% of SPANZA respondents. ESPA respondents and APAGBI respondents did not use dexmedetomidine as often: 53% and 34%, respectively (Table 1).

**Table 1:** Baseline characteristics of all respondents

	Total		ESPA		SPANZA		APAGBI		SPA	
Member	5171		849		320		872		3130	
Response rate	791	15.3%	229	27.0%	111	34.7%	129	14.8%	322	10.3%
Do you use dexmedetomidine in paediatric anaesthesiology?										
Yes	552	69.8%	121	52.8%	77	69.4%	44	34.1%	310	96.3%
No	206	26.0%	90	39.3%	32	28.8%	80	62.0%	4	1.2%
Missing	33	4.2%	18	7.9%	2	1.8%	5	3.9%	8	2.5%
Total	791	100.0%	229	100.0%	111	100.0%	129	100.0%	322	100.0%
What type of hospital do you work at?										
Tertiary	558	70.5%	152	66.4%	91	82.0%	93	72.1%	222	68.9%
Paediatric	116	14.7%	22	9.6%	4	3.6%	13	10.1%	77	23.9%
Secondary	87	11.0%	38	16.6%	12	10.8%	18	14.0%	19	5.9%
Primary	21	2.7%	10	4.4%	4	3.6%	4	3.1%	3	0.9%
Missing	9	1.1%	7	3.1%	0	0.0%	1	0.8%	1	0.3%
Total	791	100.0%	229	100.0%	111	100.0%	129	100.0%	322	100.0%
What percentage of your work includes paediatric anaesthesiology?										
10%	44	5.6%	23	10.0%	8	7.2%	8	6.2%	5	1.6%
25%	74	9.4%	25	10.9%	14	12.6%	11	8.5%	24	7.5%
50%	88	11.1%	37	16.2%	11	9.9%	5	3.9%	35	10.9%
75%	169	21.4%	45	19.7%	41	36.9%	40	31.0%	43	13.4%
100%	402	50.8%	90	39.3%	36	32.4%	63	48.8%	213	66.1%
Missing	14	1.8%	9	3.9%	1	0.9%	2	1.6%	2	0.6%
Total	791	100.0%	229	100.0%	111	100.0%	129	100.0%	322	100.0%
What type of anaesthesiologist are you?										
Paediatric	693	87.6%	182	79.5%	97	87.4%	112	86.8%	302	93.8%
Ped training	29	3.7%	11	4.8%	5	4.5%	5	3.9%	8	2.5%
General	43	5.4%	23	10.0%	8	7.2%	7	5.4%	5	1.6%
Resident	7	0.9%	3	1.3%	0	0.0%	1	0.8%	3	0.9%
Missing	19	2.4%	10	4.4%	1	0.9%	4	3.1%	4	1.2%
Total	791	100.0%	229	100.0%	111	100.0%	129	100.0%	322	100.0%

### Respondents who use dexmedetomidine

Overall, 70% of the respondents used dexmedetomidine in paediatric practice. A protocol was not available for members of ESPA (57/121, 47%) and SPANZA (33/77, 43%), whereas nearly all SPA members (285/310, 92%) had access to a protocol, as well as the majority of the APAGBI members (27/44, 61%). The drug was used for children of all ages: SPA members used dexmedetomidine mainly in patients younger than 1 year of age, whereas all other respondents used dexmedetomidine mainly in patients older than 1 year of age. Overall, dexmedetomidine was mostly used for procedural sedation (375/552, 68%), as reported by ESPA respondents (78/121, 65%) and SPA respondents (253/310, 82%). The main indication for members of SPANZA (62/77, 81%) and members of APAGBI (30/44, 68%) was premedication.

Broad ranges of dosages were reported for the use of dexmedetomidine for different applications (Table 2). For premedication, the most frequently used dose was 2.0 μg/kg intranasal bolus administration. For procedural sedation, intensive care sedation, anaesthesia and postoperative analgesia an intravenous infusion was the most frequently used route of administration. Dosages for intravenous administration differed widely (Table 2). Oral and intramuscular administration was reported as well.

**Table 2:** Dexmedetomidine dosages reported by respondents (median with interquartile ranges, minimum and maximum dose)

Setting	Bolus min ug kg^−1^	Bolus max ug kg^−1^	IV min mg kg/h^−1^	IV max mg kg/h^−1^
Premedication (nasal) Min–max	2.00 [1.00–2.00] 0.00–5.00	2.00 [2.00–3.00] 0.20–5.00	1.50 [0.50–4.00] 0.25–5.00	2.50 [1.00–4.25] 0.50–5.00
Procedural sedation Min–max	1.00 [0.50–2.00] 0.00–8.00	1.10 [1.00–2.38] 0.20–8.00	0.50 [0.30–1.00] 0.00–2.50	1.0 [0.70–1.75] 0.20–6.00
Intensive care sedation Min–max	1.00 [0.50–1.00] 0.50–8.00	1.0 [0.50–1.05] 0.50–8.00	0.50 [0.30–0.70] 0.00–5.00	1.0 [0.70–1.50] 0.10–7.00
Anaesthesia Min–max	0.50 [0.50–1.00] 0.00–2.00	1.00 [0.50–1.00] 0.00–4.00	0.50 [0.30–0.70] 0.00–5.00	1.00 [0.50–1.00] 0.03–4.00
Postoperative analgesia Min–max	0.50 [0.40–0.50] 0.00–2.00	0.50 [0.50–1.00] 0.20–3.00	0.40 [0.20–0.50] 0.00–1.00	0.50 [0.43–1.00] 0.10–2.00
Other Min–max	0.50 [0.30–1.00] 0.10–2.00	0.85 [0.50–1.00] 0.20–5.00	0.90 [0.50–1.75] 0.10–2.00	1.0 [0.78–2.00] 0.60–3.00

Median [IQR] and minimum and maximum (min–max) dosage administered. Lowest and highest reported doses for bolus and continuous infusion of dexmedetomidine. All settings, except for premedication, was mainly administered intravenously

**Fig. 2:**
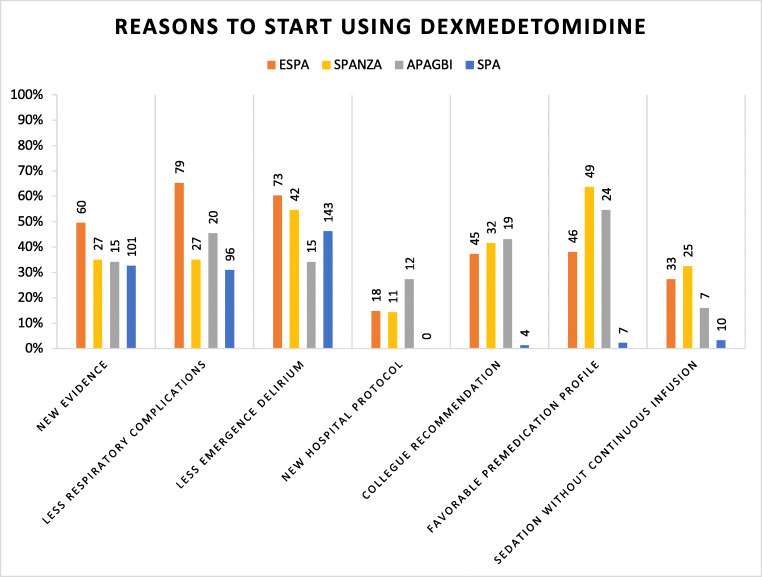
Reasons why respondents started using dexmedetomidine. Multiple answers possible

The arguments to start using dexmedetomidine in paediatric practice were fewer cases of emergence delirium compared to traditionally used anaesthetics (273/552, 50%) and fewer respiratory complications (222/552, 40%). The SPA respondents mainly reported fewer emergence delirium cases (143/310, 46%), the ESPA respondents fewer respiratory complications (79/121, 65%). The APAGBI respondents (24/44, 55%) and SPANZA respondents (49/77, 64%) mainly reported a good profile for premedication (Fig. 2). Few respondents had started using dexmedetomidine for its opioid-sparing effect. Fifty-six respondents reported they had received specific training or training through a protocol. Others had individually consulted relevant literature (*n* = 98); had discussed the application with colleagues (*n* = 79) or had learned to use dexmedetomidine via trial and error (*n* = 50).

The most commonly observed adverse drug reactions were bradycardia (*n* = 129) and nausea (*n* = 99). However, many respondents from all societies (275/552, 50%) had not observed adverse drug reactions. Respondents from the SPA were not familiar with contraindications (286/310, 92%), but the majority of respondents from other societies (67%) were aware of contraindications for the use of dexmedetomidine in paediatric care (Table 3).

**Table 3:** Responses of respondents who use dexmedetomidine

	Total		ESPA		SPANZA		APAGBI		SPA	
	552		121		77		44		310	
Is there a protocol available in your centre?										
Yes. A protocol is available	402	72.8%	57	47.1%	33	42.9%	27	61.4%	285	91.9%
No. No protocol is available	102	18.5%	47	38.8%	37	48.1%	11	25.0%	7	2.3%
Missing	48	8.7%	17	14.0%	7	9.1%	6	13.6%	18	5.8%
Total	552	100.0%	121	100.0%	77	100.0%	44	100.0%	310	100.0%
In what age categories do you use dexmedetomidine?*										
- 0–3 months old	363	65.8%	50	41.3%	23	29.9%	6	13.6%	284	91.6%
- 3 months to 1 year	404	73.2%	76	62.8%	37	48.1%	17	38.6%	274	88.4%
- 1 to 4 years	330	59.8%	100	82.6%	63	81.8%	34	77.3%	133	42.9%
- 4 to 6 years	291	52.7%	102	84.3%	73	94.8%	38	86.4%	78	25.2%
- 6 to 12 years	388	70.3%	87	71.9%	70	90.9%	35	79.5%	196	63.2%
- Older than 12 years	256	46.4%	75	62.0%	63	81.8%	33	75.0%	85	27.4%
For what purposes do you use dexmedetomidine?*										
For premedication	255	46.2%	57	47.1%	62	80.5%	30	68.2%	106	34.2%
For procedural sedation	375	67.9%	78	64.5%	34	44.2%	10	22.7%	253	81.6%
For IC sedation	251	45.5%	65	53.7%	17	22.1%	10	22.7%	159	51.3%
For anaesthesia	93	16.8%	31	25.6%	40	51.9%	13	29.5%	9	2.9%
For postoperative analgesia	178	32.2%	27	22.3%	11	14.3%	6	13.6%	134	43.2%
What adverse drug reactions have you experienced?^a^										
Hypotension	78	14.1%	46	38.0%	19	24.7%	6	13.6%	7	2.3%
Hypertension	20	3.6%	16	13.2%	1	1.3%	1	2.3%	2	0.6%
Bradycardia	129	23.4%	74	61.2%	29	37.7%	11	25.0%	15	4.8%
Hypoxia	55	10.0%	2	1.7%	0	0.0%	0	0.0%	53	17.1%
Apnoea	9	1.6%	6	5.0%	1	1.3%	1	2.3%	1	0.3%
Nausea	99	17.9%	0	0.0%	0	0.0%	0	0.0%	99	31.9%
Emergence delirium	16	2.9%	13	10.7%	3	3.9%	0	0.0%	0	0.0%
None	275	49.8%	27	22.3%	36	46.8%	22	50.0%	190	61.3%
Are you familiar with any contraindications for the use of dexmedetomidine?										
Yes. I am familiar with contraindications	170	30.8%	88	72.7%	52	67.5%	23	52.3%	7	2.3%
No. I am not familiar with contraindications	350	63.4%	25	20.7%	22	28.6%	17	38.6%	286	92.3%
Missing	32	5.8%	8	6.6%	3	3.9%	4	9.1%	17	5.5%
Total	552	100.0%	33	100.0%	25	100.0%	21	100.0%	303	100.0%
How would you rate your experience with dexmedetomidine? Median [IQR]										
			8 [7–9]		8 [7–8]		8 [7–8]		10 [9–10]	

^*^Multiple answers possible

### Respondents who do not use dexmedetomidine

**Fig. 3:**
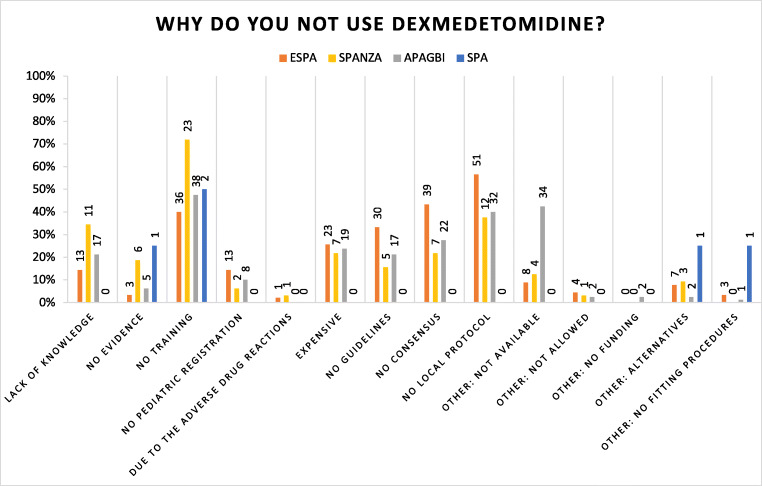
Reasons why respondents do not use dexmedetomidine. Multiple answers possible

In total, 206 of the 791 respondents (26%) did not use dexmedetomidine in paediatric care, mainly members of ESPA (90/229, 39%) and APAGBI (80/129, 62%) (Table 4). Most of them had not been educated in the use of dexmedetomidine (182/206, 88%) but were familiar with the drug (89/229, 49%). Lack of education was one of the main reasons not to use dexmedetomidine in paediatric practice (99/206, 48%). Other perceived barriers to using dexmedetomidine were the absence of local protocol (95/206, 46%) and no consensus amongst local staff (68/206, 33%) (Fig. 3). Furthermore, for 46 of these 206 respondents (22%), the drug was not available, mainly reported by APAGBI respondents (34/80, 43%). The majority of non-users (174/206, 85%) were willing to start using dexmedetomidine for premedication (126/174, 72%) and for procedural sedation (133/174, 76%, Table 4). The most important reasons for the willingness to start using dexmedetomidine were the benefits (82/174, 47%) and the safer alternative to the currently used drugs (38/174, 22%). Reasons for not being willing to start using dexmedetomidine (29/206, 14%) were the availability of better alternatives (7/29, 24%), no need (9/27, 31%) and need for more individual and general experience with dexmedetomidine (7/29, 24%).

**Table 4:** Responses of respondents who do not use dexmedetomidine

	Total 206		ESPA 90		SPANZA 32		APAGBI 80		SPA 4	
Are you trained in the use of dexmedetomidine?										
Yes	23	11.2%	8	8.9%	4	12.5%	9	11.3%	2	50.0%
No	182	88.3%	81	90.0%	28	87.5%	71	88.8%	2	50.0%
Missing	1	0.5%	1	1.1%	0	0.0%	0	0.0%	0	0.0%
Total	206	100.0%	90	100.0%	32	100.0%	80	100.0%	4	100.0%
Are you familiar with dexmedetomidine?										
Yes	89	48.9%	43	53.1%	18	64.3%	26	36.6%	2	100.0%
No	90	49.5%	37	45.7%	10	35.7%	43	60.6%	0	0.0%
Missing	3	1.6%	1	1.2%	0	0.0%	2	2.8%	0	0.0%
Total	182	100.0%	81	100.0%	28	100.0%	71	100.0%	2	100.0%
Are you willing to start using dexmedetomidine?^a^										
Yes. I am willing to start using	174	84.5%	77	85.6%	29	90.6%	66	82.5%	2	50.0%
Yes: good alternative	38	21.8%	14	15.6%	8	25.0%	16	20.0%	0	0.0%
Yes: benefits of the drug	82	47.1%	31	34.4%	13	40.6%	36	45.0%	2	50.0%
Yes: literature information	4	2.3%	3	3.3%	0	0.0%	1	1.3%	0	0.0%
Yes: if recommended	3	1.7%	2	2.2%	0	0.0%	1	1.3%	0	0.0%
Yes: if more information is available	7	4.0%	3	3.3%	1	3.1%	3	3.8%	0	0.0%
Yes: other	40	23.0%	24	26.7%	7	21.9%	9	11.3%	0	0.0%
Total	174		77		29		66		2	50.0%
No. I am not willing to start using	29	14.1%	12	13.3%	3	9.4%	12	15.0%	2	50.0%
No: better alternative	7	24.1%	5	5.6%	0	0.0%	1	1.3%	1	25.0%
No: not registrated in country	4	13.8%	1	1.1%	1	3.1%	2	2.5%	0	0.0%
No: no protocols	0	0.0%	0	0.0%	0	0.0%	0	0.0%	0	0.0%
No: no need	9	31.0%	3	3.3%	2	6.3%	3	3.8%	1	25.0%
No: price	1	3.4%	1	1.1%	0	0.0%	0	0.0%	0	0.0%
No: more experience needed	7	24.1%	2	2.2%	0	0.0%	5	6.3%	0	0.0%
No: other	1	3.4%	0	0.0%	0	0.0%	1	1.3%	0	0.0%
Total	29	100.0%	12	98.9%	3	100.0%	12	97.5%	10	100.0%
For what purposes would you use dexmedetomidine?^a^										
For premedication	126	72.4%	42	54.5%	26	89.7%	58	87.9%	0	0.0%
For procedural sedation	133	76.4%	65	84.4%	20	69.0%	48	72.7%	0	0.0%
For IC sedation	58	33.3%	34	44.2%	7	24.1%	17	25.8%	0	0.0%
For anaesthesia	75	43.1%	27	35.1%	11	37.9%	37	56.1%	0	0.0%
For postoperative analgesia	48	27.6%	18	23.4%	8	27.6%	22	33.3%	0	0.0%
Missing	1	0.6%	0	0.0%	0	0.0%	0	0.0%	1	50.0%
Other	8	4.6%	4	5.2%	2	6.9%	1	1.5%	1	50.0%

^a^Multiple answers possible

## Discussion

This international survey revealed that despite the off-label use in children, dexmedetomidine is frequently used in paediatric anaesthesiology settings, even without the availability of national or local protocols. The main indications were premedication, procedural sedation and IC sedation. Most of the anaesthesiologists who used dexmedetomidine reported the absence of adverse drug reactions, as well as awareness of contraindications for the use in paediatrics.

A prospective pilot study showed that dexmedetomidine-based anaesthesia creates satisfactory conditions for paediatric surgery [4]. Dexmedetomidine proved to be useful for various types of surgical procedures, such as airway procedures, neurosurgery, cardiac surgery and ambulatory procedures [18]. Dexmedetomidine has not been approved for use in paediatric care in any country worldwide, which would explain why structured education of paediatric anaesthesiologists on its use by manufacturers is lacking. Only 56 respondents (10%) who use dexmedetomidine had received such education. Most respondents did not have access to structured education and taught themselves by reading scientific papers, discussing with colleagues and/or were self-taught. Respondents from the SPA primarily used dexmedetomidine in patients younger than 1 year of age, whereas respondents from other societies mainly used it in patients older than 1 year of age. This discrepancy might be related to the interpretation of the 2016 FDA statement concerning the effects of anaesthesia on the young brain, i.e. children younger than 3 years of age [19]. Shortly thereafter, a consensus statement for European anaesthesiologists concluded that there was no compelling evidence to change anaesthetic practice, but that unnecessary procedures should be avoided [20]. These conflicting statements may have had effects on the change of current practice. Since anaesthesiologists in the USA have been warned for the effects on the young brain, it is likely that they would search for a less neurotoxic alternative to the traditional anaesthetics and use this alternative in the young patients. This may also explain why respondents who do not use dexmedetomidine are willing to start using it as an alternative to the currently used anaesthetics, as dexmedetomidine is thought not to be neurotoxic and provides satisfying sedation. Other different indications reported by the respondents from the different societies cannot be explained by the issued warnings. SPANZA and APAGBI members used dexmedetomidine mainly for premedication, whereas ESPA and SPA members used it mainly for procedural sedation. In the present survey study, the opioid-sparing effect was another reason for some anaesthesiologists to start using dexmedetomidine in paediatric practice. Administration of dexmedetomidine intraoperatively has been associated with a lesser need for postoperative analgesia and a lesser need for fentanyl intraoperatively [21–24].

Our survey showed that dosing regimens for paediatric care differ widely. Studies describing the pharmacokinetics of dexmedetomidine indicate that children would require a higher dosage per kilogram bodyweight compared to adults to achieve comparable exposure, due to a larger volume of distribution in children [25]. A previous study described the off-label use of dexmedetomidine in paediatric care within the European Union (EU), indicated for ICU sedation, anaesthesia and procedural sedation [7]. In the EU, a maximum infusion rate of 1.4 μg kg^−1^ h^−1^ is approved for adult sedation; however, a recently published study in Europe showed that infusion rates exceeding 1.4 μg kg^−1^ h^−1^ were used in 11% of children [7]. Our study shows a similar practice: reported infusion rates ranged from 0.1 to 3.0 μg kg^−1^ h^−1^. Dosing seems to vary inter-individually, especially in paediatrics, which may largely be the result of clearance maturation with age and consequently changing elimination half-life [15]. Furthermore, the dosing regimen depends on the route of administration. For nasal administration, a 2 μg kg^−1^ dose was the most commonly used premedication dose in the present study. Therefore, various dosing regimens should be available for physicians to use.

Dexmedetomidine pharmacodynamics and pharmacokinetics have mainly been described in adult populations. Despite the fact that several papers have described the PK of dexmedetomidine in infants over the last years [26–29], there still is an urgent need for well-designed studies describing the PK as well as the PD of dexmedetomidine in infants covering all age groups following intravenous and nasal administration. Given the large variability of estimated PK parameters between paediatric and adults populations, such as the estimated volume of distribution and the clearance which changes rapidly in paediatric patients under the age of 1 year, more evidence is needed. Reported studies suggest that this inter-individual variability is substantially larger than the effect of maturation alone. The variability may be also influenced by the variety of processes involved in metabolism and excretion of inactive dexmedetomidine metabolites following glucuronidation, methylation as well as oxidation by CYP-enzymes which are renally excreted. Especially for children under 1 year of age, pharmacokinetics and pharmacodynamics are less known [15].

Bradycardia and hypotension were the most frequently reported side effects of dexmedetomidine. Generally, these side effects do not require additional treatment [15]. Interestingly, only SPA respondents reported an adverse drug reaction, which was nausea. A meta-analysis has shown that dexmedetomidine prevents postoperative nausea and vomiting in children and in adults when administered during general anaesthesia [30]. The SPA respondents mostly use dexmedetomidine in patients younger than 1 year of age and respondents from the other societies mostly in patients above 1 year of age, which might explain the difference in reported adverse drug reactions [31].

Dexmedetomidine is not the only anaesthetic drug used off-label in paediatric anaesthesiology. Other studies have found that most drugs administered to induce and to maintain anaesthesia are off-label [32]. Our study confirmed that dexmedetomidine is used in infants, the age group with the least number of drugs licenced for use [33]. In a previous study, patients treated with off-label drugs had a significantly higher risk of adverse drug reactions [34]. As we should not expose children to unnecessary risks, it is important to investigate the pharmacokinetics and pharmacodynamics of new drugs in clinical trials in the paediatric population [35]. In the absence of trials, formal education for those prescribing and administering dexmedetomidine to children would be necessary.

Our survey revealed various barriers to the use of dexmedetomidine in paediatric practice. The main barriers were the price of dexmedetomidine, non-availability of the drug, the lack of knowledge and the lack of education. The introduction of new drugs or adjusted use of drugs in anaesthesia rarely comes with the education of the anaesthesiologists [36]. However, bed-side teaching of an anaesthesiologist with experience (local opinion leader) in the use of dexmedetomidine during anaesthesia could counteract this barrier, because this is an intervention for successful implementation [37, 38]. Unfortunately, our survey did not investigate the reason why the lack of training has such a negative effect on the use of dexmedetomidine, specifically. Mainly respondents from the APAGBI reported not to have access to dexmedetomidine, which explains the low use amongst APAGBI members. This might be due to the fact that dexmedetomidine has not been licenced for anaesthesia in the UK [39, 40]. Another reason why respondents do not use dexmedetomidine is that they lack information about the drug. Long-term effects on the use of dexmedetomidine in children have not yet been published. Studies have focused on the short-term effects, such as safety of administration, emergence delirium, postoperative nausea and vomiting [41].

The use of dexmedetomidine amongst respondents from different societies clearly differed with regard to patient age categories, routes of administration, bodyweight-based dosages, dosing regimens and the availability of protocols. We argue that paediatric anaesthesiologists from different societies must learn from each other’s experiences, share information and ultimately reach a consensus on the optimal dexmedetomidine therapy in paediatric anaesthesia. Consensus could be reached by evidence, expert interpretation and experience. Consequently, appropriate use of dexmedetomidine would be stimulated and lead to fewer differences in drug prescriptions amongst hospitals and consequently improve patient safety [42]. This consensus should also include adverse drug reactions in infants and provide an option to report suspected adverse drug events for pharmacovigilance. According to the published data in 2020 in the public dashboard of FDA Adverse Events Reporting System (FAERS), currently, 16% (269/1698) of the reports regarding dexmedetomidine as a suspect agent for an adverse event were found in the paediatric population [43]. Of these 269 reports, 247 are classified as serious. These data support the need for close monitoring of patients, adequate pharmacovigilance, more information on the PK/PD in paediatrics and awareness of potential life-threatening events in off-label use.

We hypothesise that the unproven neurotoxicity of currently used anaesthetics reduces the need for the introduction of new drugs. The lack of evidence on the advantages of dexmedetomidine does not provide a reason to change current practice by introducing a new off-label drug with unknown short-term and long-term risks.

Although we reached out to four major societies and associations for paediatric anaesthesiologists, we could not reach all paediatric anaesthesiologists because not everybody is a member of one of these societies. We probably missed a large proportion, primarily those working in Asia, Africa and South America [44]. E-mails with a link to the survey were sent out by the societies themselves, on different dates after July 16, 2019. The closing date was the same for all societies: August 16. The different time windows to respond to the survey could have led to response bias. We acknowledge the responder and non-responder bias for this survey, which could have influenced the results. Those who do not use dexmedetomidine in paediatrics might have been less likely to participate since they do not have any benefits from participation in the study. The target response rate was set at 25%, based on the anaesthesiologists’ heavy workload. The total number of anaesthesiologists approached could be an overestimation because some anaesthesiologists may a member of multiple societies, which could be an explanation of the low response rate. Furthermore, except for ESPA members, we do not know how many anaesthesiologists actually read the e-mailed invitation and did not respond or how many anaesthesiologists missed the e-mail because it was relegated to the “spam” folder. Furthermore, since physicians receive at least one survey daily, it is not likely that they participate in every survey [45].

The majority of respondents in this survey use dexmedetomidine in paediatric anaesthesia, despite its off-label status and the lack of protocols. Dexmedetomidine provides sedation with minimal respiratory depression and a quick onset mechanism. Furthermore, it quickly wears off, augments analgesia and is associated with only mild adverse drug reactions that rarely require treatment. Intercontinental sharing of experience and information would be desirable. Due to the off-label use and lack of evidence on dexmedetomidine in children, a consensus amongst experts on the use of this promising drug is decisive for future use. Peer-reviewed protocols, dosage recommendations and teaching opportunities would be helpful in sharing the promising properties and safety of dexmedetomidine in paediatric care.

## Data Availability

The data that supports the findings of this study are available in the article and the supplementary material of this article.

## Supplementry Information

ESM 1 (DOCX 22 kb).
