# Supplementary material for: Off-label use of dexmedetomidine in paediatric anaesthesiology: an international survey of 791 (paediatric) anaesthesiologists
Source: Eur J Clin Pharmacol. 2020 Oct 29;77(4):625–35. doi: 10.1007/s00228-020-03028-2 (PMC7935836; doi:10.1007/s00228-020-03028-2)
Supplement: ESM 1 — (DOCX 22 kb). [file 228_2020_3028_MOESM1_ESM.docx]

Supplementary Table 1. Online survey questions and answers

| **Question** | **Answers** |
| --- | --- |
| 1. What country do you practise medicine at? | Albania, Algeria, Andorra, Angola, Armenia, Austria, Australia, Argentina, Azerbaijan, Belarus, Belgium, Benin, Bosnia and Herzegovina, Bolivia, Bulgaria, Burkina Faso, Burundi, Canada, Cameroon, Chad, Chile, Croatia, Colombia, Congo, Cote d'Ivoire, Cyprus, Czech Republic, Denmark, Ecuador, Egypt, Eritrea, Estonia, Ethiopia, Finland, France, French Guiana, Gabon, Gambia, Georgia, Germany, Ghana, Greece, Guinea, Guyana, Hungary, Iceland, Ireland, Israel, Italy, Kazakhstan, Kenya, Kosovo, Latvia, Libya, Liberia, Liechtenstein, Lithuania, Luxembourg, Macedonia (FYROM), Madagascar, Malta, Malawi, Mali, Morocco, Mexico, Moldova, Monaco, Montenegro, Mozambique, Netherlands, New Zealand, Niger, Nigeria, Norway, Paraguay, Peru, Poland, Portugal, Qatar, Romania, Russia, San Marino, Saudi Arabia, Serbia, Senegal, Sierra Leone, Slovakia, Slovenia, Somalia, South Africa, Spain, Sudan, Suriname, Sweden, Switzerland, Tanzania, Togo, Turkey, Uganda, Ukraine, United Kingdom (UK), Uruguay, USA, United Arabian Emirates, Vatican City, Venezuela, Zambia, Zimbabwe |
| 1. What kind of hospital do you work at? | - Tertiary/university (paediatric) hospital |
|  | - Paediatric hospital |
|  | - General hospital (secondary referral) |
|  | - General hospital (rural/primary) |
|  | - Other |
| 1. What amount of your (clinical)   work comprises paediatric  anaesthesia? | - 10% |
|  | - 25% |
|  | - 50% |
|  | - 75% |
|  | - 100% |
| 1. Do you have a special training in paediatric anaesthesia? | - Specialized paediatric anaesthesiologist (with or without fellowship) |
|  | - Currently in training for paediatric anaesthesiologist |
|  | - General anaesthesiologist |
|  | - Resident anaesthesiology |
| 1. How many years have you been working as an anaesthesiologist (after registration)? | In years |
| 1. Do you use dexmedetomidine   in paediatric practice? | - Yes |
|  | - No |
| IF NOT USING DEXMEDETOMIDINE IN PAEDIATRIC PRACTICE | |
| 1. Are you trained in the use of dexmedetomidine in paediatric setting? | - Yes |
|  | - No |
| 1. Are you familiar with dexmedetomidine? | - Yes |
|  | - No |
| 1. Why do you not use dexmedetomidine? | - Lack of knowledge about dexmedetomidine in general |
|  | - Lack of evidence of dexmedetomidine in paediatric care |
|  | - Lack of personal experience/no training for dexmedetomidine use in paediatric setting |
|  | - Not registered for use in paediatric population |
|  | - Negative side effects/Adverse events |
|  | - Too expensive |
|  | - No national/international guideline available for paediatric use |
|  | - No consensus among local staff |
|  | - No local protocol |
|  | - Other |
| 1. Are you willing to start working with dexmedetomidine in paediatric setting? | - Yes, because |
|  | - No, because |
| 1. If previous question is YES:   For what purposes are you willing to use dexmedetomidine? | - Premedication (for a non-invasive procedure/anaesthesia/etc.) |
|  | - Procedural sedation |
|  | - Intensive care sedation |
|  | - General anaesthesia |
|  | - Postoperative analgesia |
|  | - Other |
| 1. Are you interested in participating in a multicentre prospective study regarding use of dexmedetomidine in paediatric anaesthesia? If yes, please fill in your e-mail address | - Yes, my e-mail address is: |
|  | - No |
| IF USING DEXMEDETOMIDINE IN PAEDIATRIC PRACTICE | |
| 1. Is there a protocol available at your centre for dexmedetomidine use in paediatric setting? | - Yes |
|  | - No |
| 1. What age are the patients you use dexmedetomidine for? | - Neonates (age 0 - 3 months) |
|  | - Baby (age 3 months - 1 year) |
|  | - Toddler (age 1 - 4 years) |
|  | - Child (age 4 - 6 years) |
|  | - Scholar (age 6 - 12 years) |
|  | - Teenager (age > 12 years) |
| 1. What do you use dexmedetomidine for? Please provide dose used in ug/kg for bolus and mcg/kg/hr for continuous infusion | - Premedication (ug/kg) |
|  | - Procedural sedation (ug/kg) |
|  | - Intensive care sedation (ug/kg) |
|  | - Anaesthesia (ug/kg) |
|  | - Postoperative analgesia (ug/kg) |
|  | - Other (ug/kg) |
| 1. What was the reason to start using dexmedetomidine in paediatric care? | - New evidence |
|  | - No respiratory complications |
|  | - Less emergency delirium |
|  | - New hospital protocol |
|  | - Colleague recommendation |
|  | - Good profile as premedication (not irritating, no bad taste) |
|  | - Practical use for sedation without continuous infusion |
|  | - Other |
| 1. How long have you been using dexmedetomidine for in paediatric setting? | - 1-6 months |
|  | - 6-12 months |
|  | - 1-2 years |
|  | - >2 years |
| 1. How were you trained in using dexmedetomidine in paediatric setting? | Open question |
| 1. Have you experienced any clinically relevant adverse events when using dexmedetomidine? | - Hypotension |
|  | - Hypertension |
|  | - Bradycardia |
|  | - Hypoxia |
|  | - Apnoea |
|  | - Nausea |
|  | - Agitation/emergence delirium |
|  | - None |
|  | - Other |
| 1. Are you familiar with any contraindications for the use of dexmedetomidine in paediatric setting? | - No |
|  | - Yes, namely: |
| 1. How would you rate your overall experience with dexmedetomidine use in paediatric care on a scale from 1 to 10? | Mean grade |
| 1. Are you interested in participating in a multicentre prospective study regarding use of dexmedetomidine in paediatric anaesthesia? If yes, please fill in your e-mail address | - No |
|  | - Yes, my e-mail address is: |
